# Better Choice, Better Health? Social Integration and Health Inequality among International Migrants in Hangzhou, China

**DOI:** 10.3390/ijerph17134787

**Published:** 2020-07-03

**Authors:** Xiaoguang Fan, Fei Yan, Wei Yan

**Affiliations:** 1 Department of Sociology, Zhejiang University, Hangzhou 310058, China; xgfan@zju.edu.cn; 2 Department of Sociology, Tsinghua University, Beijing 100084, China; 3 Department of Psychology, Tsinghua University, Beijing 100084, China; yanw17@mails.tsinghua.edu.cn

**Keywords:** self-rated health, self-assessed change in health, immigrant, China, social integration

## Abstract

The aim of this study is to investigate the impact of social integration and socioeconomic status on immigrant health in China. Taking the framework of social determinants of health (SDH) as the theoretical starting point, this paper uses the Hangzhou sample of the 2018 Survey of Foreigners in China (SFRC2018) to explore two core factors affecting the health inequality of international migrants in China: the level of social integration following settlement, and socioeconomic status before and after coming to China. The results show that having a formal educational experience in China helped improve both the self-rated health status and self-assessed change in health of international migrants; that the socioeconomic status of an emigrant’s home country affected self-rated health; and that the self-assessed change in health of immigrants from developing countries was significantly higher than those from developed countries. This study concludes that the health inequalities of immigrant populations in China must be understood in the context of China’s specific healthcare system and treatment structure.

## 1. Introduction

International migration has always been an important global issue, closely related to the political, economic, and social development of society. International migration in China, the world’s second largest economy, has attracted widespread attention from the Chinese government and academia. The sixth census showed that in 2010 there were 593,800 foreign residents from 129 countries and regions in China who came for business, employment, study, and settlement [1]. The sample included foreigners and residents of Hong Kong, Macao, and Taiwan who had resided in or were expected to reside in mainland China for more than three months; those living in mainland China for shorter periods on business or through tourism were not included. Based on the author’s calculations, of those sampled, 134,900 (22.72%) came for employment; because there is no strict distinction between the working-age population and the non-working-age population, there is some theoretical underestimation of these statistics. (See http://www.stats.gov.cn/tjsj/pcsj/rkpc/6rp/indexch.htm.) The United Nations estimated that one million foreigners were living in China in 2017, an increase of 22,000 from 2015 [2]. The immigrant population in China today is no longer limited to businesspeople from high-income countries, but also includes a growing number of people from low- and middle-income countries.

However, thus far there has been a dearth of research on immigrant health in China. Previous studies focused either on internal migration and health at the provincial level [3,4,5,6,7] or on health conditions of Chinese immigrants to other industrial countries [8,9,10,11,12]. Relatively little has been done to analyze the impact of social integration and socioeconomic status on immigrant health in China. The only immigrant subgroups that have been studied are the African immigrants in Guangzhou, where there are now at least 20,000 legal African residents, and an unknown number of illegal residents residing in the city, making Guangzhou home to Asia’s largest African migrant population [13,14]. Based on an in-depth qualitative study, scholars have found that African immigrants in Guangzhou experienced various barriers to accessing health care and were generally dissatisfied with local health services [15,16].

To fill this research gap, this paper used the Hangzhou sample of the 2018 Survey of Foreigners in China (SFRC2018)—the first quantitative survey of the health status and health-service perceptions of international migrants in China—to explore the influence of pre- and post-immigration circumstances on immigrant populations’ self-rated health status and self-assessed changes in health. We chose Hangzhou because, as an e-commerce hub in China, Hangzhou is one of the favorite destinations for international migrants. According to the Hangzhou Health and Family Planning Commission, the foreign resident population in the city in 2017 was about 20,000. Compared with Guangzhou, foreigners living in Hangzhou are more diversified, coming mainly from the United States, South Korea, Germany, and from countries in Africa and South America [17].

Theoretically, we take the framework of social determinants of health (SDH) as the theoretical starting point. The SDH framework demonstrates that a person’s health is governed by three layers of elements: individual lifestyle system (gender, age, race, and constitutional factors); social/community networks; and general socioeconomic, cultural, and environmental conditions [18,19,20,21]. For international migrants facing the challenges of adjusting to a new country—language, job skills, cultural orientation, social inclusion, and access to medical resources—immigration itself is a crucial health factor [22,23,24].

In particular, we examined two core factors affecting the health inequality of immigrant populations in China: socioeconomic status before and after coming to China, and social integration following settlement. Moreover, we also considered the issue of health inequality within China’s current healthcare structure and its institutional constraints on the supply of public-health resources to international migrants. Our results demonstrate that having a formal educational experience in China helps promote immigrant integration and adaptation to the local society and therefore improves both self-rated health status and self-assessed change in health of international migrants; that the socioeconomic status of an emigrant’s home country affects self-rated health; and that immigrants from developing countries tend to perceive more improved health status than those from developed countries.

## 2. Social Determinants of Health

The most fundamental causes of health inequalities are related to different socioeconomic conditions [25,26,27,28]. According to the World Health Organization, “The social determinants of health are the conditions in which people are born, grow, live, work and age. These circumstances are shaped by the distribution of money, power and resources at global, national and local levels [29]”.

Within the framework of SDH, social integration and support are vital factors. Scholarship on European and American international migrants has shown that social integration contributes to improved health [30,31,32]. High levels of social integration relate positively to immigrant health via two mechanisms. First, having a wide local network leads to more local friends, better understanding of the local culture, and increased informal instrumental or emotional support. For example, a longitudinal study of Korean immigrants to Canada found that ethnic social support from one’s neighborhood community tends to ameliorate psychological distress [33]. Another study showed that local educational experiences may contribute to lesser feelings of perceived social distance from mainstream society [34]. Second, having a good command of language leads to better access to, and higher utilization skills for obtaining and understanding, local medical and health resources. Previous research has reported that language barriers are associated with less understanding of basic health information and less satisfaction with health services [35]. Addressing language barriers improves the communication between patients and providers and thereby increases access to health care.

Socioeconomic status is another primary influencer on immigrant population health. Studies have found that almost all societies have different degrees of socioeconomic gradient of health, whereby the higher the socioeconomic position of people and communities, the better the health status [36,37,38,39]. In other words, people of different socioeconomic status have significant differences in average health levels. Unlike education and occupation, socioeconomic status is particularly related to health care, health insurance, and access to diversified health services. Socioeconomic status determines the relative ability to acquire resources in a specific institutional environment. Groups with high status tend to receive superior health care services and to access pre- and post-treatment with less health risks. Groups with lower status are relatively disadvantaged in the distribution of medical resources because the medical system provides only basic coverage for disadvantaged groups.

For the immigrant population, the country of origin is considered an important determinant in explaining the differences in health status and health-service utilization [40,41,42]. Studies have shown that in the 1970s most immigrants from developing countries, such as those from Latin America and Asia, had a significantly higher prevalence of infectious diseases than did native residents in the United States [43,44]. Another strand of immigration study has reported an immigrant health negative effect in which many health indicators, including mortality, heart disease, and obesity, of immigrants from developing countries are higher than those of the native-born populations in the destination countries [45,46].

The specific medical and treatment structure of China’s health care system is significantly different from those in Europe and the United States. Most of China’s high-quality health care resources are concentrated in public hospitals rather than in private and international hospitals. Institutions offering direct international health services are still relatively rare and extremely expensive in China [47,48,49]. The lack of professional medical interpretation or translation services at the organizational level creates an additional “lost in translation” barrier to accessing health care service. Especially noteworthy are the challenges of translation in traditional Chinese medicine, which greatly limits immigrants’ medical treatment options in China [50]. As such, international migrants often require help from Chinese friends to navigate the public hospital–centered system or are compelled to seek medical services from other channels [23].

Moreover, most immigrants in China come from low- and middle-income developing countries, and the medical resources they encounter in China tend to be better than those in their home countries. Generally, immigrants entering countries with a more optimized distribution system of medical resources are more likely to evaluate their health status positively than immigrants entering countries with less optimized health care service systems due to a relative sense of satisfaction.

From the above discussions, we can observe that the level of social integration and socioeconomic status have an explanatory power for health inequality. In this article, we aim to explore such effects among immigrant populations in Hangzhou, China. Since international migrants often face pressure from all sides in the destination country, how do adaptation to the local environment and changed socioeconomic status affect immigrants’ health status, and to what extent does this pattern vary by different country type?

## 3. Data, Variables, and Methods

### 3.1. Data and Sample

The data used in this study come from the 2018 Survey of Foreigners in China (SFRC2018) in which the authors’ research group had participated extensively in the data collection work. The project, established in 2016 by the Institute of State Governance of Sun Yat-sen University, expanded from the initial Guangzhou survey in 2016–2017 to include six more cities in 2018: Hangzhou, Xi’an, Changchun, Lanzhou, Yiwu, and Xuzhou [23]. In particular, the Hangzhou subsample was collected by the authors’ research group at Zhejiang University in July and August of 2018 for four weeks. SFRC2018 was the first comprehensive social survey of the health status and health care service perceptions of foreigners in China since the sixth census in 2010, whose subjects also included those from Hong Kong, Macao, and Taiwan. The survey was conducted via questionnaire administered onsite at the primary exit-entry administration office of different cities. Participants were recruited through a convenience sampling method. Each recruited participant was briefed about research aims and data confidentiality policy and were requested to voluntarily participate by signing consent forms. Apart from collecting data on participants’ sociodemographic characteristics, including gender, age, education level, current marital status, and job status in China, the survey also asked about foreigners’ immigration experience, labor and employment, social mentality, social life, and health care utilization. Relevant ethical approval and informed consent were obtained before the survey.

A total of 1132 questionnaires were collected for the Hangzhou subsample. After careful examination, 1034 valid questionnaires were obtained, and the effective response rate reached 91.34%. After strict data cleansing, the sample size of foreigners entering our analysis was 932. (Because the highest percentage of missing values for all variables was 3.48%, we did not use multiple interpolation (MI) as a remedy. We directly deleted missing cases.)

### 3.2. Variables

The dependent variable in this paper was health inequality, which refers to the subjective evaluation of the individual’s physical health, including the two dimensions of self-rated health and self-assessed change in health. Self-rated health refers to a single question in the survey: “In general, would you say that your health is excellent, very good, good, fair, or poor?” Only 6.54% of the respondents indicated that their health was “fair” or “poor.” The respondents who regarded their health as “excellent” reached 61.63%. We assigned *excellent* a value of 1, and *very good and below* a value of 0.

Though the existing literature has widely shown that self-rated health has robust predictive power on mortality, morbidity, and individuals’ physical functioning [51,52,53], this measure might still be problematic since it primarily reflects an individual’s subjective evaluation of his or her own health and therefore might be affected by ceiling effects, where people reporting the highest level of health tend not to report subsequent improved health. An alternative way of assessing change in health is to ask respondents whether their health has become better or worse over time [54,55]. As such, in the present analysis we also included self-assessed change in health as our dependent variable.

Self-assessed change in health emphasizes an immigrant’s perception of health change before and after immigration to China. In the survey, we asked a health transition question: “How do you rate your health now since you came to China?” This variable was assigned five values: *much worse*, *a litter worse*, *same*, *a litter better*, and *much better*. For our analysis, we consolidated these five values into two—we merged the first two scales into *becoming worse* (=0) and the last three items into *same/becoming better* (=1).

Our core independent variables included social integration, socioeconomic status, and country type. Social integration was measured by the educational experience of studying in China. Having a formal educational experience in the country of immigration has a significant effect on an immigrant’s level of social integration. Studying in Chinese universities or primary and secondary schools helps international migrants develop a well-rounded understanding of Chinese culture and society and builds strong social networks with native residents. We created a dummy variable with reference to *not studying in China* (=0). Meanwhile, we believe that language proficiency, especially fluency in spoken Chinese, enables immigrants to feel more comfortable with and adept at seeking medical treatment and that it would therefore be a mediating mechanism for the impact of social integration on health status. In the survey, language proficiency was measured at four levels: *proficient*, *good*, *understanding a little*, and *completely unfamiliar*, and we combined *understanding a little* and *completely unfamiliar* as the reference group *little or none* (=0).

Socioeconomic status was measured by two variables—respondent’s socioeconomic status in their home country, and current occupational status in China. The socioeconomic status in the home country was measured by a five-degree subjective self-evaluation: *very high*, *high*, *middle*, *low*, and *very low*. We combined the last three scales into *middle and below* (= 0) as our reference group. Further, in the questionnaire the current occupational status was divided into seven categories: (1) businessperson; (2) manager, professional specialty, technician; (3) white collar and sales; (4) craftspeople and repair workers; (5) unskilled workers (excluding farm workers); (6) servants and service workers; and (7) farm laborers. We further divided these occupations into four groups: I, *self-employed* (1); II, *employed* (2–7); III, *unemployed* (nonstudent); and IV, *students*, with *students* as the reference group (=0)

Country types were classified into three categories, according to the World Bank’s classification criteria: high-income, middle-income, and low-income countries, with reference to *low-income countries* (=0). (Please refer to https://data.worldbank.org/country.)

In addition to the aforementioned core independent variables, we also included multiple control variables. We used gender and marital status as dummy variables, with *male* (=0) and *unmarried* (=0) as reference groups. In addition, the length of stay in China (unit: month) and education (*no university degree* = 0) are also possible influential factors on the health status of immigrants (Chang and Wallace, 2016). The length of stay in China was operated as 0–12, 13–36, 37–60, ≥61 months. Age was also included as a continuous control variable in the model.

### 3.3. Analytic Strategies

In this study, both self-rated health and self-assessed change in health are dichotomous non-continuous variables. Therefore, the binary logit model was used. To overcome the bias caused by missing variables, we also used the bivariate probit model to assess the robustness of statistical results [56]. Because the two dependent variables in this research depend on the same list of independent variables and are correlated, we employ the bivariate probit model which implements the maximum likelihood method to fit the model of the binary choice with binary endogenous regressors. When analyzing the internal mechanism, we used the Karlson-Holm-Breen (KHB) method to decompose the effects in non-linear probability models [57]. All of the statistics in this paper are produced in STATA 16.1.

## 4. Results

**Figure 1:**
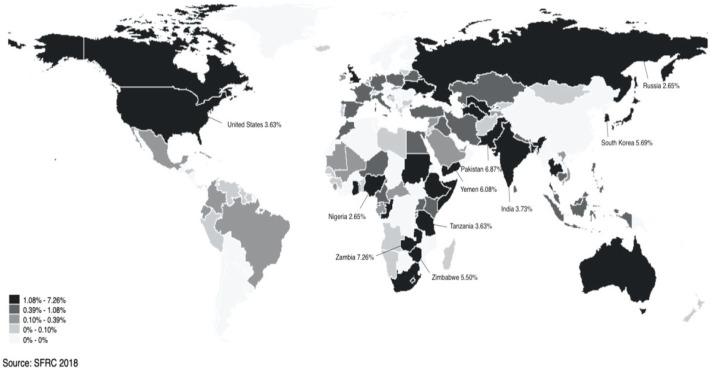
National origins of immigrants in Hangzhou (2018).

Figure 1 shows the national origins of our surveyed respondents of the SFRC2018 in Hangzhou. They came from 112 countries and regions, with Zambia, Pakistan, Yemen, South Korea, and Zimbabwe as the top five largest sending countries. Table 1 further shows the descriptive statistics of the main variables. The results show that more than 60% (61.6%) of the foreigners surveyed said that they were in excellent health; 85.3% of the foreigners surveyed thought that their health condition had either stayed the same or had improved after coming to China, while less than 15% (14.7%) of the foreigners surveyed thought that their health condition had worsened. With respect to socioeconomic status, the status of foreigners in their home country was generally good, with 37.6% belonging to the high level of socioeconomic status and more than 10% (10.9%) belonging to the very high level. In the current occupational structure, nearly 70% (67.3%) of surveyed respondents were international students. The percentage of foreigners in business activities was close to 10% (9.2%), and 11.30% of surveyed foreigners were unemployed. In terms of country type, only 22.9% came from high-income countries. In addition, the foreigners surveyed had a high level of education, with more than 90% (91.6%) having a university degree. The average age was less than 30 years old (mean = 26.98, sd = 8.36). In general, these sociodemographic characteristics largely replicate those of the Guangzhou survey [23].

In addition to the above variable distribution, we also performed a chi-square test on the core variables and dependent variables (see Table 2). In terms of the dimension of social integration, the proportion of foreigners who were proficient in Chinese and self-rated that they were in excellent health was 70.29%; of those who knew a little Chinese or hardly understood Chinese, 60.61% thought that they were in excellent health. The chi-square test is only significant at the level of 0.1. In contrast, Chinese proficiency did not lead to significant differences in changes of self-assessed health. In terms of the experience of studying in China, the self-rated health and self-assessed change in health of those who had studied in China were significantly higher than those who had not.

With regard to country type and health inequality, respondents from high-income countries reported lower levels of health (*p* < 0.000) for both self-rated health and self-assessed change in health. In terms of the status dimension, socioeconomic status in the home country and occupational status in China had different effects on the two dependent variables of health inequality. The socioeconomic status of foreigners in their home country had a significant positive effect on self-rated health status, but it did not affect self-assessed change in health. In terms of occupational status in China, there were significant differences in self-rated health status of different categories. All of the above statistics were based on cross-tabulation analysis, and we needed to control more variables to reach an accurate conclusion.

Table 3 shows a binary logistic regression analysis of the self-rated health of foreigners in Hangzhou. Among them, Model 1 includes the experience of studying in China, and Model 2 includes language proficiency based on Model 1. Model 3 includes country type, and Model 4 includes socioeconomic status of the respondents in the home country. Model 5 includes occupational status in China, and Model 6 also includes professional status in China and socioeconomic status of the home country. After controlling for gender, age, marital status, education level, and length of stay in China, the self-rated health status of those who had studied in China was significantly higher than that of foreigners who had not studied in China. When we controlled for language skills, studying in China still had a significant effect. Meanwhile, the results in the full model were basically the same.

In Model 3, foreigners from middle-income countries (MICs) and high-income countries (HICs) had lower self-rated health than foreigners from low-income countries (LICs) (*p* < 0.01, *p* < 0.001). This shows that when controlling for other variables, the effect of macroscopic resource distribution on the self-rated health of foreigners is supported by data.

As shown in Model 4, socioeconomic status in the home country had a significant impact on self-rated health. Even if employment status in China (Model 6) is controlled, social status in China did not have the same effect (Model 5). Compared with foreigners from middle and lower socioeconomic statuses, the probability of a high-status person having an *excellent healthy* self-evaluation was increased by 51.1%, and for those of very high status, it was increased by 283.8%. This shows that the role of socioeconomic status in the home country was significant. The above effects did not change significantly in the whole model, and our findings are consistent with previous studies in which scholars have found that the socioeconomic status experienced in childhood has a lasting impact on future health [58].

Next, we performed the binary logistic regression analysis of the self-assessed change in health status of foreigners in Hangzhou (see Table 4). Among them, the model setting was completely consistent with Table 3. In terms of social integration, the experience of studying in China (Model 1) had a significant impact on self-assessed change in health, but Chinese-language proficiency level showed a significant inhibitory effect (Model 2), which was different from Model 2 in Table 3. The country-type effect in Model 3 was the same as the self-rated health status, which means that self-assessed change in health (*same/becoming better*) of foreigners from high-income countries was significantly lower than for those in low-income countries. Similarly, in terms of socioeconomic status, the socioeconomic status in the home country had a significant impact on self-assessed change in health, which was significantly different from self-rated health measure (Model 4 in Table 3). Finally, the socioeconomic status in China had no significant effect on the self-assessed change in health status of foreigners.

To assess the robustness of the above results, we introduced bivariate probit regression to overcome the endogeneity problems caused by missing variables. In Table 5, the dependent variable of Model 1 is self-rated health, and the dependent variable of Model 2 is self-assessed change in health status. By comparison, we found that in addition to educational experiences and country type showing consistent stabilizing effects in the two models, the effects of other factors on the dependent variables were inconsistent.

Finally, we looked at how social integration affected the health inequalities of immigration populations in China. Table 6 examines whether language proficiency was a mediating mechanism for social integration that affected the health inequality of foreigners. Regarding self-rated health status, Chinese language proficiency had no significant impact on the explanatory power of studying in China. However, in terms of self-assessed change in health, the difference between the reduced model and the full model was significant. This shows that the experience of studying in China can affect the health inequality of foreigners through a command of Chinese language.

## 5. Discussions

This study made a first attempt to report immigrants’ perceived health status and evaluate health change in China, but there are still several limitations that deserve further improvement. First, the convenience sampling method of participants in the study might impose some inherent selection bias. This potential bias of the sampling technique due to under-representation of subgroups in the sample in comparison to the population of interest might lead to too little variance in the main variables of interest (SES). Given the lack of sufficient variance in SES, it is not surprising that we find no significant association between socioeconomic status in China and self-assessed change in health status of foreigners. Second, student participants were overrepresented in the sample, and therefore our findings do not convey differences in health service use according to social rank groups. Third, since our research is based on quantitative analysis, a qualitative research of immigrant’s health behaviors and practices would be useful to further explore the health issues and health experiences among immigrant populations in China. Fourth, is Hangzhou case representative of the immigrant population in China? As Wilkinson and Pickett pointed out, health is less good in societies where income differences are bigger [59]. In essence, the income gap between rich and poor reflects the scale of social class differences in a society, making the poor feel more hostile and more depressed. The essence of social stratification is the distribution of valuable resources, which inevitably leads to different degrees of relative deprivation. The stronger the relative deprivation of individuals, the larger the mental stress individuals will face and the more protracted the periods of experiencing negative emotions, thereby increasing the risk of chronic illnesses. Along this line of thinking, combining resource distribution analysis at the macro level with social inequality case studies at the micro level, and exploring the formation mechanism of different dimensions of health, will be useful to further study of immigrant health inequality, not only in Hangzhou, but also in other places in China.

## 6. Conclusions

In this paper, we used the latest dataset on immigrants in China and employed a SDH framework as a theoretical basis to examine the impact of social integration and socioeconomic status on immigrant populations’ health status in Hangzhou, China. In the analysis, we also considered the specific cultural and institutional structure of China’s medical system and its implications for immigrants.

Our results show that, first, having had an educational experience in China not only helped improve the self-rated health status of international migrants, but also had a positive effect on self-assessed change in health status. This finding is consistent with our expectations. Educational experiences in China provided immigrants with a deeper understanding of both the overall health care system in China and the unique characteristics of Chinese medical diagnostic and treatment protocols. However, the mediating effect of language ability did not affect health status significantly. A speculative explanation is that although language skills include speaking, writing, and listening, in our survey we investigated only the level of proficiency in spoken Chinese. This limitation is therefore not conducive to a comprehensive evaluation of the level of immigrants’ social integration.

Second, the higher the socioeconomic status of immigrants in their country of origin, the higher their self-rated health status. Meanwhile, the self-assessed change in health of immigrants from low- and middle-income countries was significantly higher than that of immigrants from developed countries. We believe that for most immigrants from developing countries, China’s medical system and the country’s supply capacity of public resource are relatively superior to those in their home countries; even if they could not take advantage of China’s public resources, immigrants in China still favorably compared Chinese resources to those of their home countries.

## Figures and Tables

**Table 1:** Descriptive statistics (*n* = 932).

Variables	Percentage	Frequency
**Health outcomes**		
Self-rated health status		
Very good and below	38.40	358
Excellent	61.60	574
Self-assessed change in health		
Becoming worse	14.70	137
Same/Becoming better	85.30	795
**Social determinants**		
Study in China		
Yes	69.60	649
No	30.40	283
Chinese proficiency		
Little or none	50.50	471
Good	36.20	337
Proficient	13.30	124
Employment status		
Self-employed	9.20	86
Employed	12.20	114
Unemployed	11.30	105
Students	67.30	627
SES in home country		
Middle and below	51.50	480
High	37.60	350
Very high	10.90	102
Country of Origin		
LICs	25.30	236
MICs	51.80	483
HICs	22.90	213
**Sociodemographic variables**		
Gender		
Male	68.70	640
Female	31.30	292
Marital status		
Married	23.40	218
Never married	76.60	714
Education		
College and beyond	91.60	854
High school and below	8.40	78
Length of stay		
0–12 months	28.00	261
13–36 months	31.30	292
37–60 months	25.60	238
≥60 months	15.10	141

**Table 2:** Cross-tabulation between social determinant and health categories.

Variables	Self-Rated Health Status			Self-Assessed Change in Health		
	Very Good and Below	Excellent	χ^2^	Becoming Worse	Same/Becoming Better	χ^2^
Study in China			χ^2^ = 29.371 (*p* = 0.000)			χ^2^ = 4.619 (*p* = 0.032)
Yes	32.81	67.19		12.72	87.28	
No	50.30	49.70		17.79	82.21	
Chinese proficiency			χ^2^ = 5.103 (*p* = 0.078)			χ^2^ = 2.703 (*p* = 0.259)
Little or none	39.39	60.61		12.62	87.38	
Good	40.17	59.83		15.77	84.23	
Proficient	29.71	70.29		17.16	82.84	
Employment status			χ^2^ = 16.985 (*p* = 0.001)			χ^2^ = 8.788 (*p* = 0.032)
Self-employed	45.74	54.26		21.98	78.02	
Employed	44.26	55.74		14.75	85.25	
Unemployed	50.79	49.21		19.47	80.53	
Students	34.30	65.70		12.43	87.57	
SES in home country			χ^2^ = 31.14 (*p* = 0.000)			χ^2^ = 0.642 (*p* = 0.725)
Middle and below	45.19	54.81		14.62	85.38	
High	35.50	64.50		13.62	86.38	
Very high	17.59	82.41		16.67	83.33	
Country of Origin			χ^2^ = 24.08 (*p* = 0.000)			χ^2^ = 18.65 (p = 0.000)
LICs	26.00	74.00		7.72	92.28	
MICs	40.37	59.63		14.48	85.52	
HICs	46.67	53.33		21.65	78.35	

**Table 3:** Logit regression analyses of self-rated health among international migrants.

Variables	Model 1	Model 2	Model 3	Model 4	Model 5	Model 6
Constant	0.335	0.414	1.233 ***	0.640	0.926 **	0.619
	(0.373)	(0.378)	(0.355)	(0.348)	(0.341)	(0.351)
Study in China (*yes* = 1)	0.673 ***	0.685 ***				
	(0.165)	(0.170)				
Chinese proficiency (ref. *little/none*)						
Good		−0.263				
		(0.162)				
Proficient		0.363				
		(0.249)				
Country of Origin (ref. *LICs*)						
MICs			−0.577 **			
			(0.180)			
HICs			−0.759 ***			
			(0.217)			
SES in home country (ref. *middle and below*)						
High				0.413 **		0.403 **
				(0.148)		(0.148)
Very high				1.345 ***		1.319 ***
				(0.285)		(0.285)
Employment status (ref. *students*)						
Self-employed					−0.319	−0.242
					(0.256)	(0.259)
Employed					-0.199	−0.131
					(0.223)	(0.227)
Unemployed					−0.470 *	−0.409
					(0.234)	(0.238)
Control variables	Yes	Yes	Yes	Yes	Yes	Yes

* p < 0.05, ** *p* < 0.01, *** *p* < 0.001. All models also control for age, gender, marital status, education, and duration.

**Table 4:** Logit regression analyses of self-assessed change in health among international migrants.

Variables	Model 1	Model 2	Model 3	Model 4	Model 5	Model 6
Constant	2.013 ***	2.177 ***	2.928 ***	2.624 ***	2.492 ***	2.556 ***
	(0.546)	(0.554)	(0.557)	(0.522)	(0.518)	(0.527)
Study in China (*yes* = 1)	0.642 **	0.754 ***				
	(0.220)	(0.229)				
Proficiency in Mandarin (ref. *little/none*)						
Good		−0.446 *				
		(0.224)				
Proficient		−0.361				
		(0.318)				
Country of Origin (ref. *LICs*)						
MICs			−0.664 *			
			(0.278)			
HICs			−1.171 ***			
			(0.310)			
SES in home country (ref. *middle and below*)						
High				0.010		−0.006
				(0.205)		(0.206)
Very high				−0.355		−0.405
				(0.298)		(0.300)
Employment status (ref. *students*)						
Self-employed					−0.663 *	−0.691 *
					(0.318)	(0.320)
Employed					−0.175	−0.194
					(0.303)	(0.304)
Unemployed					−0.364	−0.384
					(0.306)	(0.307)
Control variables	Yes	Yes	Yes	Yes	Yes	Yes

* p < 0.05, ** *p* < 0.01, *** *p* < 0.001. All models also control for age, gender, marital status, education, and duration.

**Table 5:** Bivariate probit regression between self-rated health status and self-assessed change in health.

Variables	Model 1		Model 2	
	Coef.	S.D.	Coef.	S.D.
Study in China (*yes* = 1)	0.376 **	(0.121)	0.334 *	(0.138)
Chinese ability (ref. *little/none*)				
Good	−0.139	(0.102)	−0.221	(0.120)
Proficient	0.280	(0.148)	−0.158	(0.175)
Country of Origin (ref. *LICs*)				
MICs	−0.316 **	(0.111)	−0.271	(0.142)
HICs	−0.342 *	(0.142)	−0.567 ***	(0.170)
SES in home country (ref. *middle and below*)				
High	0.248 **	(0.093)	−0.017	(0.113)
Very high	0.775 ***	(0.162)	−0.304	(0.168)
Employment status (ref. *students*)				
Self-employed	0.153	(0.178)	−0.074	(0.198)
Employed	0.112	(0.152)	0.105	(0.181)
Unemployed	−0.100	(0.161)	−0.037	(0.184)
Control variables	Yes	-	Yes	-
Constant	0.263	(0.256)	1.514 ***	(0.316)

* p < 0.05, ** *p* < 0.01, *** *p* < 0.001. All models also control for age, gender, marital status, education, and duration.

**Table 6:** Decomposition of effects in logit models for study in China.

**Self-Rated Health Status (Logit)**					
	**Coef.**	**s.e.**	***p***	**95% Conf. Interval**	
Reduced	0.682	0.165	0.000	0.357	1.006
Full	0.685	0.170	0.000	0.352	1.019
Diff	−0.004	0.043	0.934	−0.088	0.081
**Self-Assessed Change in Health (Logit)**					
	**Coef.**	**s.e.**	***p***	**95% Conf. Interval**	
Reduced	0.652	0.221	0.003	0.220	1.084
Full	0.754	0.229	0.001	0.305	1.202
Diff	−0.102	0.057	0.074	−0.213	0.010

All models also control for age, gender, marital status, education, and duration.
